# A Rapid and High-Throughput Quantitation Assay of the Nuclear Factor κB Activity Using Fluorescence Correlation Spectroscopy in the Setting of Clinical Laboratories

**DOI:** 10.1371/journal.pone.0075579

**Published:** 2013-10-04

**Authors:** Kenu Harada, Shintaro Mikuni, Hideyuki Beppu, Hideki Niimi, Shigeki Abe, Nobuko Hano, Koichi Yamagata, Masataka Kinjo, Isao Kitajima

**Affiliations:** 1 Department of Clinical Laboratory and Molecular Pathology, Graduate School of Medicine and Pharmaceutical Science for Research, University of Toyama, Toyama, Japan; 2 Department of Advanced Optical Imaging Research, Research Center for Cooperative Projects, Graduated School of Medicine, Hokkaido University, Sapporo, Japan; 3 Laboratory of Molecular Cell Dynamics, Faculty of Advanced Life Science, Hokkaido University, Sapporo, Japan; 4 Sysmex Corporation BMA Laboratory, Kobe, Japan; CNR, Italy

## Abstract

**Background:**

Transcription factor nuclear factor-κB (NF-κB) plays a key role in the regulation of immune responses to inflammation. However, convenient assay systems to quantitate the NF-κB activity level in a timely manner are not available in the setting of clinical laboratories. Therefore, we developed a novel and high-throughput quantitative assay based on fluorescence correlation spectroscopy (FCS) to detect the NF-κB activity level in cellular nuclear extracts and evaluated the performance of this method. The basic principle of this assay is to calculate the binding fraction of NF-κB to fluorescent-labeled DNA probes, which contain NF-κB binding sites.

**Methods:**

Non-fluorescent competitive probes are employed to normalize the influence of the viscosity of the nuclear extracts between samples and to eliminate the influence of nonspecific binding of the fluorescent probes. To confirm accurate quantitation, human recombinant NF-κB p50 was mixed into U937 cell nuclear extracts, and the binding fraction of the fluorescent probes to NF-κB in the mixture was calculated for quantitation. To evaluate whether this method can be applied to measure the NF-κB activity in human lymphocytes, the NF-κB activity levels of systemic inflammatory response syndrome patients during perioperative periods were measured.

**Results:**

The percentage recovery was 88.9%. The coefficients of variation of the intra-assay were approximately 10%. NF-κB activity levels during the perioperative period can were successfully measured. The assay time for the FCS measurement was within 20 minutes.

**Conclusions:**

This assay system can be used to quantitate NF-κB activity levels in a timely manner in the setting of hospital laboratories.

## Introduction

Nuclear factor-κB (NF-κB) has been identified as a regulator of the expression of the κB light chain in B cells [1]. NF-κB represents a family of transcription factors that share an N-terminal Rel homology domain (RHD) responsible for homo- and hetero-dimerization, nuclear translocation, and DNA-binding [2]. In quiescent cells, dimerized NF-κBs are inactivated in the cytoplasm via interaction with inhibitory proteins, IκBs. The p65/p50 heterodimers are the primary targets of IκBα [2]. NF-κB p50 plays an important role in the immune responses to inflammation [3]. In response to inflammatory mediators such as tumor necrosis factor-α (TNF-α) and interleukin (IL)-1, IκBs are rapidly degraded via the ubiquitin–proteasome pathway, directing NF-κB to translocate into the nucleus to bind to specific DNA sequences and promote the expression of various target genes, including IL-6. This pathway is crucial for activation of inflammation and innate immunity [4]. The timely assessment of the NF-κB activity regulating inflammatory cytokines may be necessary for earlier diagnosis of acute inflammatory diseases such as systemic inflammatory response syndrome (SIRS). Rapid and high-throughput quantitative methods for detecting the DNA binding activity of NF-κB in these diseases are highly desirable.

The conventional method to measure the NF-κB activity is the electrophoretic mobility shift assay (EMSA) [5]. However, it is a laborious and time-consuming procedure that typically requires the use of radioactive probes and antibodies against NF-κBs. Luciferase reporter assays have also been used to detect the DNA binding and transcriptional activity. These assays, however, are difficult to apply in high-throughput screening of clinical samples. Renard and colleagues established a more convenient DNA binding assay on the basis of a modified enzyme-linked immunosorbent assay (ELISA) [6], [7]. However, this assay is unfit for emergency tests such as evaluating the degree of acute inflammation (e.g. systemic inflammatory response syndrome) because it requires at least three hours for the assay time.

Recently, new methods of rapid and high-throughput platforms such as fluorescence resonance energy transfer (FRET) and fluorescence correlation spectroscopy (FCS) [8]–[13] have been developed to study NF-κB-DNA interactions in the liquid phase. FCS allows for the analysis of molecular interactions and complex formation in the liquid phase at the level of a single molecule [14], [15]. In an FCS analysis, fluctuations of fluorescent intensity caused by Brownian motion of fluorescent-labeled molecules can be analyzed as the diffusion time and the number of molecules that move across a confocal detection volume (10^-15^ liter) [16]. Because the diffusion time of a molecule depends on its molecular weight, molecular interactions (between proteins and fluorescent-labeled DNA) can be evaluated according to the increased diffusion time in the solution [17].

We herein report that a modified FCS assay using non-fluorescent competitors is capable of quantitating the NF-κB activity level in various crude nuclear extracts. By using this method, the increased NF-κB activity levels induces by TNF-α treatment were measured in human lymphocytes. Moreover, increased NF-κB activity levels were observed prior to increase of IL-6 in the perioperative period. This assay system enables rapid and high-throughput quantitation of the NF-κB activity and may contribute to clinical monitoring of patients with inflammation-associated diseases.

## Methods

### Reagents

Human recombinant NF-κB p50 (hr-p50) was purchased from Promega Corporation (Madison, WI, USA). Human recombinant TNF-α was purchased from Cell Signaling Technology, Inc. (Danvers, MA, USA).

### Cell Culture

HeLa cells were cultured in MEM, supplemented with 10% fetal bovine serum (FBS), penicillin (100 U/ml), and streptomycin (100 µg/ml). U937 cells were cultured in RPMI-1640 supplemented with 10% FBS, 2 mM L-glutamine, penicillin (100 U/ml), and streptomycin (100 µg/ml).

### Patients and Materials

Written informed consent was obtained from the cancer patients in Toyama University Hospital for the collection and the use of blood samples. This study was conducted with the approval of the Ethics Committee of the University of Toyama. Human lymphocytes were isolated from peripheral blood samples using Lymphoprep (Axis-Shield PoC AS, Oslo, Norway) according to the manufacturer’s protocol. SIRS was defined as having at least 2 of the following criteria: temperature <36°C or >38°C; heart rate >90 beats/minute; respiratory rate >20 breaths/minute; PaCO_2_<32 mmHg; and white blood cell counts >12,000 cells/mm^3^, or <4,000 cells/mm^3^, or >10% immature band forms [18].

### IL-6 Determination

IL-6 levels in human plasma were measured by a sandwich enzyme-linked immunosorbent assay kit (EH2IL6; Pierce Biotechnology, Inc., Rockford, IL, USA) according to the manufacturer’s instructions.

### Preparation of Nuclear Extracts

Nuclear proteins were extracted using the Nuclear Extraction kit (Affymetrix, Santa Clara, CA, USA). Cells were collected and homogenized in 150 µl of buffer A provided by the Nuclear Extraction kit. The cells were centrifuged at 15,000 rpm for three minutes at 4°C. The nuclear pellet was then re-suspended in 40 µl of buffer B provided by the Nuclear Extraction kit. The nuclear extracts were isolated using centrifugation for three minutes at 15,000 rpm at 4°C. The protein concentrations were determined using a Bio-Rad protein assay kit, according to the manufacturer’s instructions.

### Fluorescent-labeled DNA and Competitor DNA

The fluorescent-labeled, stem-loop oligonucleotides containing a κB binding element were as follows:

5′-TAMRA-agttgaggggactttcccaggcaaaagcctgggaaagtcccctcaa ct-3′.

TAMRA; 5/6-carboxytetramethylrhodamine. The synthesized stem-loop oligonucleotides were diluted in Tris-HCl buffer (pH 7.5), denatured at 95°C for 10 minutes and annealed at 65°C for 30 minutes.

For the competition assays, the following non-fluorescent probes were used: wild-type: 5′-agttgaggggactttcccaggcaaaagcctgggaaagtcccctcaact-3′, and nonspecific: 5′-caggcgcgttttgaccat cttaaaaaagatggtcaaaacgcgcctg-3′. The NF-κB binding sites are underlined.

### Fluorescence Correlation Spectroscopy

FCS measurement was performed using the single-molecule fluorescence detection system, MF20 (Olympus Corporation, Tokyo, Japan) [13]. Standard dye and TAMRA-labeled DNA were excited by a 250 µW, 543 nm laser line. The emission was detected through 565–595 nm for 15 seconds for each sample and repeated five times. To determine the structure parameter(*s*), TAMRA (Olympus Corporation, Tokyo, Japan) as a standard was measured. The samples were added to a 384-well glass-bottomed microplate, and the measurements were obtained in a sample volume of 20 µl. Reaction mixtures containing binding buffer (50 mM Tris–HCl buffer (pH 7.5), 250 mM NaCl, 1 mM EDTA, 2.5 mM dithiothreitol and 10% glycerol), nuclear extract sample, 1 nM TAMRA-labeled NF-κB probe, 1 µg of poly (dI-dC) and competitors were mixed and further incubated at room temperature for 10 minutes.

### Quantitation of NF-κB

**Figure 1:**
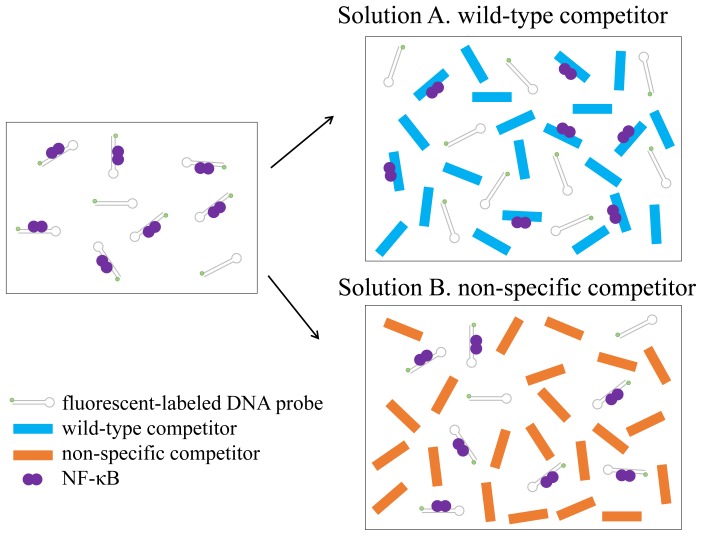
A Schematic drawing of the competition assay by FCS for the detection of the NF-κB/DNA binding activity. Solution A: This solution contains nuclear extract samples, fluorescent-labeled DNA probes and excess wild-type competitors. The diffusion time of free fluorescent-labeled DNA probes can be measured in Solution A. Solution B: this solution contains nuclear extract samples, fluorescent-labeled DNA probes and excess nonspecific competitors. The diffusion time of this solution derives from both NF-κB/DNA probe complexes and unreacted fluorescent-labeled DNA probes. The binding fraction of NF-κB-bound DNA probes to the total fluorescent-labeled DNA probes is obtained using a two-component model.



Figures 1 show schematic drawings of the competition assay based on FCS. To analyze the fraction of free and protein-bound fluorescent-labeled DNA, the fluorescence autocorrelation functions G(τ) were fitted by one- or two-component models as follows [19]:
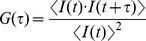


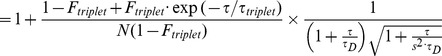
(one-component model)

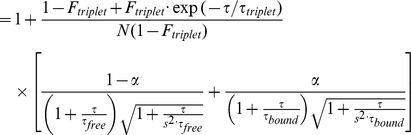
(two-component model)

where *F_triplet_* is the average fraction of triplet state molecules, *τ_triplet_* is the triplet relaxation time, *τ_D_* in the one-component model is the diffusion time, *N* is the number of fluorescent-molecules in the detection volume element defined by radius *w_0_* and length 2*z_0_* and *s* is the structural parameter representing the ratio, *s = z_0_/w_0_*.

In the two-component model, *α* is the binding fraction, which denotes the ratio of protein-bound TAMRA-labeled DNA to the total TAMRA-labeled DNA. *τ_free_* and *τ_bound_* are the diffusion times of the free and protein-bound DNA, respectively. To facilitate the quantification of the fraction of protein-bound TAMRA-labeled DNA, the values of *F_triplet_*, *τ_triplet_*, *τ_free_* and *τ_bound_* were used as fixed parameters.

### I. Generation of Standard Curves

To quantitate the concentration of endogenous NF-κB, the standard curve was generated using titration of hr-p50 before each set of experiments.

- FCS measurement was performed for the TAMRA-labeled DNA in the absence of hr-p50. The diffusion times of the free TAMRA-labeled DNA (*τ_free_*) and the triplet parameters (*F_triplet_* and *τ_triplet_*) were obtained using the one-component model.
- The diffusion time of the p50 homodimer-bound TAMRA-labeled DNA complex (*τ_bound_*) was calculated based on the *τ_free_* and the molecular weight of the complex in the Stokes-Einstein equation below. 
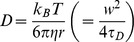
where *D* is the diffusion constant, *k_B_* is Boltzmann’s constant, *T* is the absolute temperature, *η* is viscosity, *r* is the radius of the spherical particle and is proportional to the cubic root of its molecular weight, *w* is the radius of detection volume and *τ_D_* is diffusion time.
- FCS measurement was performed for titrated hr-p50 samples as standard.
- The binding fraction (*α*) of p50 homodimer-bound TAMRA-labeled DNA to the total TAMRA-labeled DNA was obtained using the two-component model with the values of *τ_free_* and *τ_bound_* as fixed parameters.
- The value of *α* was plotted against the concentration of hr-p50.
- The standard curve for NF-κB was generated using a four parameter logistic model: 
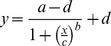
where *y* is the binding fraction of hr-p50-bound TAMRA-labeled DNA to the total TAMRA-labeled DNA, *x* is the hr-p50 concentration, *a* and *d* are the responses at zero and infinite dose (i.e., *a* = 0 and *d* = 100), *c* is the concentration giving 50% binding and *b* is the slope parameter.

### II. Analyzing the Nuclear Extract Samples

- FCS measurement was performed for the TAMRA-labeled DNA in the presence of nuclear extracts with wild-type competitors (Solution A in Figure 1). The diffusion time of the free TAMRA-labeled DNA (*τ_free-WT_*) and the triplet parameters (*F_triplet_* and *τ_triplet_*) were obtained using the one-component model.
- The *τ_bound_WT_* was calculated as described above (I-ii).
- FCS measurement was performed for the corresponding samples with nonspecific competitors (Solution B in Figure 1). The binding fraction (*α*) of protein-bound TAMRA-labeled DNA to the total TAMRA-labeled DNA was obtained using the two-component model with the values of *F_triplet_*, *τ_triplet_*, *τ_free_WT_* and *τ_bound_WT_* as fixed parameters.
- The two binding fractions [*α*(WT) and *α*(NS)] were applied to the standard curve, and the quantitative values (ng/test) were calculated.
- The value was normalized using the total amount of nuclear proteins in the sample.
- Finally, the quantitative value of p50 (ng/µg of nuclear protein) was calculated by subtracting the value of the wild-type sample from the value of the nonspecific sample.

**Figure 2:**
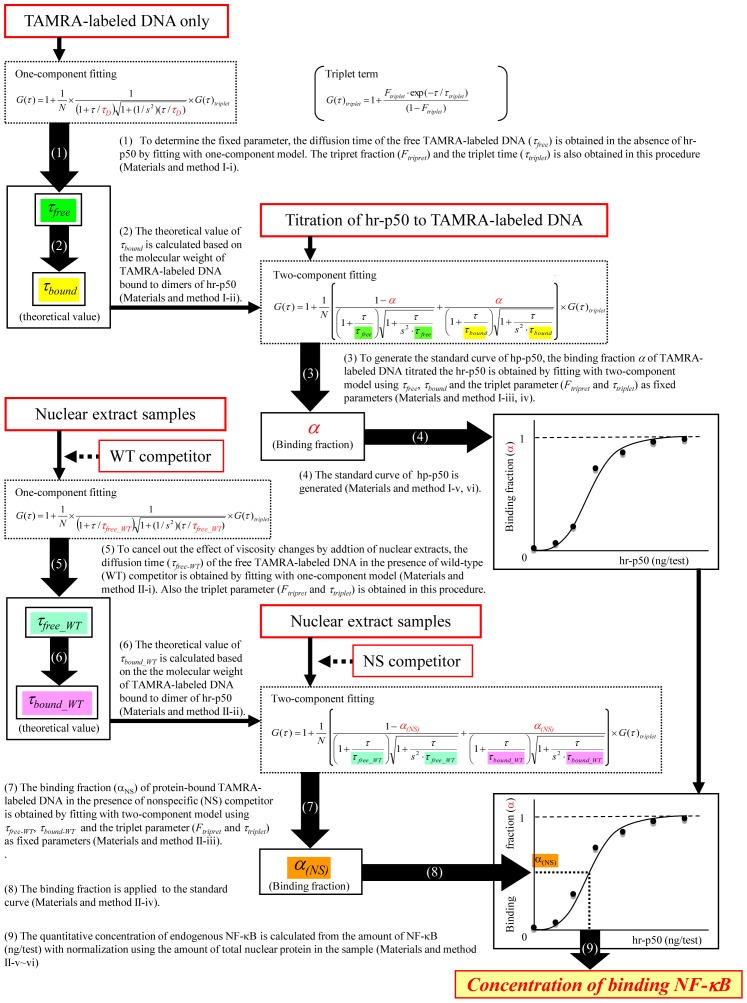
Procedures for quantitation of the NF-κB activity. The flowchart shows the procedures for quantitation of the NF-κB activity using fluorescence correlation spectroscopy with non-fluorescent competitors.

The procedures described above were summarized in Figure 2.

### Statistical Analysis

The data are expressed as the mean ± the standard error of the mean (SEM). For the statistical analyses, the groups were compared using Student’s *t*-test. *p* values less than 0.05 were considered significant.

## Results

### Quantitative Assay of the NF-κB Activity

**Figure 3:**
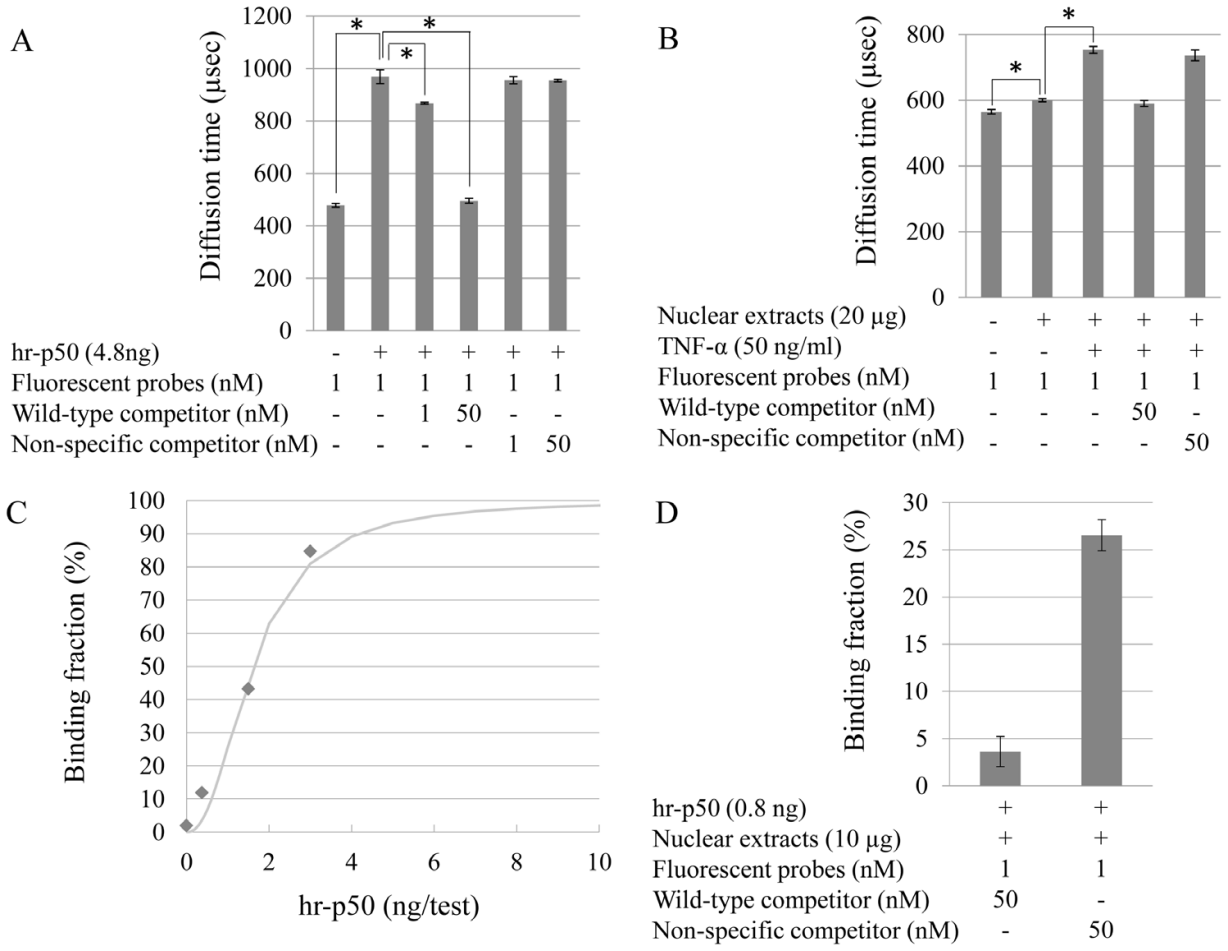
Quantitative assay of the NF-κB activity. A: The diffusion time of the NF-κB/DNA probes complex in buffer solution without nuclear extracts. Each bar represents the mean ± SEM of five measurements. *p<0.05. B: The diffusion time of the NF-κB/DNA probes complex with or without HeLa cell nuclear extracts. HeLa cells were treated with 50 ng/ml of TNF-α for 15 minutes. The values represent the mean ± SEM of five measurements. *p<0.05. C: A standard curve for quantitation of the NF-κB activity. D: The binding fraction of NF-κB-bound fluorescent-labeled probes. Each bar represents the mean ± SEM of three measurements.

The viscosity of the nuclear extracts affects the Brownian motion of the fluorescent-labeled probes, resulting in prolongation of the diffusion time when measured with FCS. To normalize the effects of varying degrees of viscosity on the diffusion time, competition assays were employed using wild-type or nonspecific competitors together with fluorescent probes. First, the diffusion time of the complex (hr-p50 and the fluorescent-labeled probes) was measured and qualitatively analyzed using one-component model in the absence or presence of wild-type competitors (Figure 3A). The diffusion time of the fluorescent probes alone was 478.2±7.5 µsec. When hr-p50 was added, the diffusion time increased (969.3±58.9 µsec) due to the complex formation of hr-p50 and the fluorescent-labeled probes. As expected, the addition of non-labeled wild-type competitors (1 nM) blocked the complex formation of the p50 and fluorescent probes and decreased the diffusion time of the complex (868.0±3.4 µsec). When an excess of wild-type competitors (50 nM) was added, the diffusion time was approximately the same as that observed in the absence of hr-p50 (495.8±9.4 µsec), indicating that the fluorescent-labeled probes were free from complex formation. In contrast, the addition of the nonspecific competitors had little effect on the diffusion time at any concentration examined (1 and 50 nM, Figure 3A), thus suggesting that complex formation is highly dependent on the specific NF-κB binding sequences of the fluorescent-labeled probes.

To evaluate the diffusion time of the complex (endogenous p50 and fluorescent probes) in the nuclear extracts, HeLa cells were treated with TNF-α (50 ng/ml) for 15 minutes, and nuclear proteins were extracted (Figure 3B). Even without stimulation by TNF-α, the diffusion time increased when the nuclear extracts were added compared to that of the fluorescent probes alone (increased from 564.8 to 600.1 µsec). These results suggest that there was a small amount of NF-κB in the TNF-α-untreated nuclei or that the viscosity of the nuclear extracts affected the diffusion time. An increased diffusion time (753.4±10.6 µsec) was observed after stimulation with TNF-α. This result indicates that TNF-α induces the complex formation of endogenous p50 with fluorescent probes. On the other hand, when the wild-type competitors were added, the diffusion time decreased to a level similar to that observed in the TNF-α-unstimulated HeLa cell nuclear extracts (590.1±9.1 µsec). This result suggests that NF-κB proteins are undetectable in TNF-α-untreated nuclei. In contrast, the nonspecific competitors did not affect the diffusion time of the TNF-α-stimulated HeLa cell nuclear extracts.

To quantitate the binding fraction, a known concentration of hr-p50 was measured without nuclear extracts to create a standard curve (Figure 3C) using two-component model, as shown in the Methods section (I i-iv). The vertical axis shows the binding fraction (%) of hr-p50-bound fluorescent probes to the total fluorescent probes. To confirm accurate quantitation with the standard curve, 0.8 ng of hr-p50 was mixed into nuclear extracts (10 µg) of U937 cells, and the binding fraction between NF-κB and the fluorescent probes in the mixture was calculated for quantitation (Figure 3D), as shown in the Methods section (II i-iv). Applying the difference between the two binding fractions with wild-type and nonspecific competitors to the standard curve, the quantitative value was 0.67 ng/test, and the percentage recovery was 88.9%.

To assess the accuracy of this method in more detail, a spike recovery test was carried out and the coefficients of variation (CV) of each intra-assay were determined. The recombinant NF-κB p50 (hr-p50) were mixed into 15 µg of U937 cell nuclear extracts. When 0.75 ng of hr-p50 was added in the nuclear extracts, the quantitative results by the FCS assay showed 0.75±0.07 ng which CV was 8.9%, and 1.66±0.18 ng which CV was 10.9% after treatment with 2.0 ng of hr-p50, respectively. These experiments were evaluated by three measurements of test samples (Table 1).

**Table 1:** Spike recovery test by the FCS assay.

Added hr-p50 (ng)	Nuclear extracts (μg)	Quantitative results (ng)	CV (%)
0.75	15	0.75±0.07	8.9
2.0	15	1.66±0.18	10.9

Quantitative tests of the recombinant NF-κB p50 (hr-p50) in 0.75 ng and 2.0 ng were performed in the presence 15 µg of U937 cell nuclear extracts. The coefficients of variation (CV) of each intra-assay were determined. Values represent the mean ± SE; n = 3 in each group.

### Quantitation of the NF-κB Activity in Isolated Human Lymphocytes by FCS Assay

**Figure 4:**
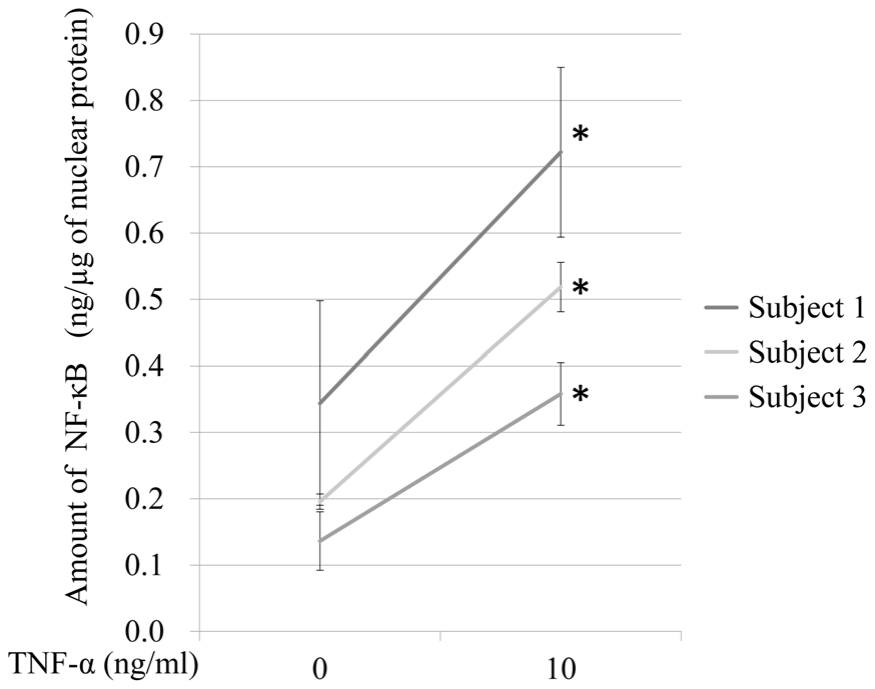
Quantitative results of the NF-κB activity in the nuclear extracts of human lymphocytes. Lymphocytes were isolated from three individuals (Subjects 1, 2 and 3) and treated with 10 ng/ml of TNF-α for 15 minutes. The NF-κB activity in the human lymphocytes was increased by TNF-α stimulation. Each bar represents the mean ± SEM of five measurements. *p<0.05.

To evaluate whether the FCS assay can be applied to measure the NF-κB activity in human lymphocytes isolated from blood, we performed a quantitative analysis of the NF-κB activity in the human lymphocytes with or without TNF-α (10 ng/ml) stimulation. The quantified amounts of NF-κB in the nuclear extracts of the human lymphocytes were detectable and increased by varying degrees in response to TNF-α stimulation (Figure 4).

### Kinetics of Activation of NF-κB by the FCS Methods in the Perioperative Period

**Figure 5:**
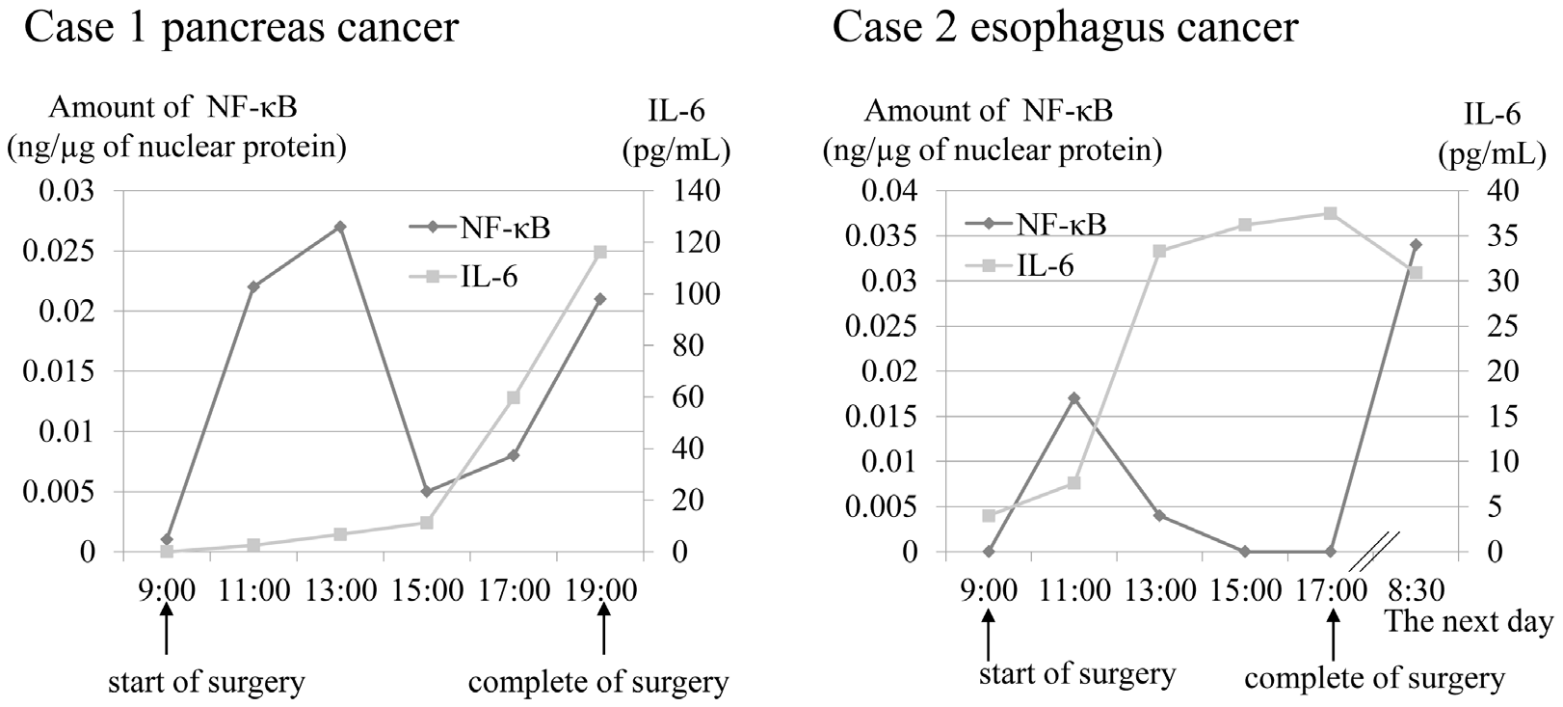
Kinetics of activation of NF-κB in the perioperative period. NF-κB activities and IL-6 were measured in the perioperative period for esophagus and pancreas cancers from two individuals.

Finally, to assess the applicability of the FCS methods in a clinical setting, we quantitate the NF-κB activity in the perioperative period of patients with cancer. In these cases, the increased NF-κB activity levels in the lymphocytes were observed 2 hours after the start of surgery (Figure 5). These increases occurred prior to increases of plasma IL-6 levels. Furthermore, in the both cases, the NF-κB activity levels increased again during or after the surgery. These patients developed SIRS after the surgery.

## Discussion

By using FCS with wild-type or nonspecific competitors, we showed that the NF-κB activity in biological samples can be easily and rapidly quantitated with a standard curve. FCS provides a powerful high-throughput technique for the detection of DNA-protein interactions in a liquid phase [13], [16], [20]–[25]. Kobayashi and colleagues reported that the NF-κB-DNA binding activity in nuclear extracts can be evaluated using the FCS method with fluorescent-labeled DNA probes [13]. Their results also showed that the diffusion time of the NF-κB/DNA probe complex is correlated with the concentration of the TNF-treated HeLa cell nuclear extracts. To date, a method for quantitation of the NF-κB activity in various samples has not been developed previously. We herein developed a novel quantitation system for detecting the NF-κB activity level using FCS with competitive probes that enables rapid and high-throughput quantitation.

Crude nuclear extracts contain various molecules, whose effects should be considered in order to quantitate the NF-κB activity. The FCS assay is known to be highly susceptible to the viscosity of the solution [15]. For instance, the diffusion coefficient of GFP in PBS was different from that in the nuclear protein [26]. As shown in Figure 3B, the diffusion time is actually susceptible to the viscosity of the nuclear extract. Therefore, to quantitate the NF-κB activity in samples with different viscosities (e.g., nuclear extracts of lymphocytes isolated from different individuals), the viscosity of each sample should be normalized. Without taking the viscosity into consideration, the amount of NF-κB in the sample solution cannot be quantitated using a standard curve made with buffer-diluted hr-p50 solutions, which have a different viscosity from the sample solutions. To normalize the influence of the viscosity, a wild-type competitor was used. In the presence of the wild-type competitor, the fluorescent-labeled DNA was released from NF-κB (Figure 1). Therefore, the amount of endogenous NF-κB in the nuclear extracts was able to be quantified according to the binding fraction obtained from measurement in the presence of a nonspecific competitor. To obtain the binding fraction from the formula based on the two-component model, the diffusion times of both free fluorescent probes and the p50-fully-bound fluorescent probes must be measured as fixed parameters. Therefore, we divided the samples into two groups and added the wild-type competitors and nonspecific competitors, respectively. Of note, the viscosity of the solution containing wild-type competitors is equal to that of the solution containing nonspecific competitors. The diffusion time obtained using the one-component model in the solution containing the wild-type competitors is assumed to be the same as the diffusion time of the free fluorescent probes (*τ_free_WT_*), as shown in Figure 3B. The diffusion time of the p50-fully-bound fluorescent probes is calculated using the Stokes-Einstein equation based on the *τ_free_WT_* (*τ_bound_WT_*). Therefore, using the two-component model, the binding fraction of the p50-bound fluorescent probes in the solution containing nonspecific competitors (i.e., the sample nuclear extracts) can be obtained. Hence, the binding fraction can be applied to a standard curve made with buffer-diluted hr-p50 solution (Figure 3C and 3D).

In the presence of the wild-type competitor, the minimal binding fractions of protein-bound fluorescent-labeled DNA were calculated (Figure 3D). This result may indicate nonspecific binding of fluorescent-labeled DNA. To normalize the nonspecific binding, subtracting the value of the wild-type sample from the nonspecific sample is required (see Methods). All NF-κB components bound to κB consensus sequences are expressed as the DNA binding activity of p50 homodimers in our FCS-based method. To demonstrate that the quantitation of NF-κB by the FCS-based method correlates with the NF-κB signaling activity, we confirmed that the inhibitor of IκB-α phosphorylation (BAY11-7082), which can disrupt the nuclear translocation of NF-κB, blocked the increase in the diffusion time induced by PMA/ionomycin (data not shown).

As shown in Table 1, the spike recovery study showed the CVs of each intra-assay were determined to be approximately 10%. We determined that these values were within acceptable ranges, because good accuracy for the determination of most clinical laboratory tests aimed at less than 10% of CV. These results indicate that the FCS-based method properly reflects the NF-κB signaling activity and that FCS is applicable for the quantitation of the NF-κB activity.

Double-strand DNA probes conjugated with TAMRA, which were formed by mixing sense and anti-sense oligonucleotides, were used as fluorescent-labeled probes in a previous report [13]. We designed new stem-loop structure DNA probes that efficiently form a double-strand structure within the molecule. The stem-loop structure probes exhibit similar sensitivity to the double-strand DNA probes mentioned above (data not shown), however, the stem-loop structure probes can be easily prepared.

Recently, ELISA-based NF-κB activity assays have been used in place of conventional methods because these methods are faster and more sensitive than EMSA [6], [27]–[29]. Whereas these assays are based on the binding of proteins on a solid surface, the novel method using FCS is based on reactions in a physiological liquid phase. The FCS method requires no specific antibodies for which the conditions must be optimized. Moreover, the assay time of the FCS method is less than 20 minutes for each sample, whereas the ELISA-based assays require several hours.

NF-κB plays a central role in coordinating the expressions of a variety of inflammatory cytokines that control immune responses. Mokart and colleagues reported that IL-6 could be an early marker of postoperative SIRS in patients undergoing major surgery for cancer [30]. We were able to quantitate the NF-κB activity level in human lymphocytes isolated from peripheral blood, showing the increase induced by TNF-α stimulation (Figure 4). Furthermore, we were able to quantitate the increased NF-κB activity levels prior to the increase of plasma IL-6 levels in the perioperative period of major surgery for cancer. These results suggest that assessment of NF-κB activity using the FCS method leads to earlier diagnosis of SIRS (Figure 5). The increased NF-κB activity levels with severe surgical stress might induce SIRS. Swiftness of measurement is one of the advantages of this method, which may aid the diagnosis of acute inflammatory diseases. The strategy used in our study is also applicable to the quantitation of activities of certain types of transcription factors, thereby making it possible to perform more precise quantitation.

In conclusion, this novel assay is a fast, sensitive and high-throughput assay for the quantitation of the NF-κB activity. This system could be useful for promptly evaluating and monitoring various inflammatory diseases in clinical laboratory settings.

## References

[1] Sen R, Baltimore D (1986) Multiple nuclear factors interact with the immunoglobulin enhancer sequences. Cell 46: 705–716. 3091258 10.1016/0092-8674(86)90346-6

[2] Hayden MS, Ghosh S (2008) Shared principles in NF-κB signaling. Cell 132: 344–362. 18267068 10.1016/j.cell.2008.01.020

[3] Ghosh S, May MJ, Kopp EB (1998) NF-κB and Rel proteins: evolutionarily conserved mediators of immune responses. Annu Rev Immunol 16: 225–260. 9597130 10.1146/annurev.immunol.16.1.225

[4] Baker RG, Hayden MS, Ghosh S (2011) NF-κB, inflammation, and metabolic disease. Cell Metab 13: 11–22. 21195345 10.1016/j.cmet.2010.12.008 PMC3040418

[5] Schreck R, Zorbas H, Winnacker EL, Baeuerle PA (1990) The NF-κB transcription factor induces DNA bending which is modulated by its 65-kD subunit. Nucleic Acids Res 18: 6497–6502. 2174540 10.1093/nar/18.22.6497 PMC332601

[6] Renard P, Ernest I, Houbion A, Art M, Le Calvez H, et al (2001) Development of a sensitive multi-well colorimetric assay for active NF-κB. Nucleic Acids Res 29: E21. 11160941 10.1093/nar/29.4.e21 PMC29628

[7] Benotmane AM, Hoylaerts MF, Collen D, Belayew A (1997) Nonisotopic quantitative analysis of protein-DNA interactions at equilibrium. Anal Biochem 250: 181–185. 9245437 10.1006/abio.1997.2231

[8] Altevogt D, Hrenn A, Kern C, Clima L, Bannwarth W, et al (2009) A new assay format for NF-κB based on a DNA triple helix and a fluorescence resonance energy transfer. Org Biomol Chem 7: 3934–3939. 19763295 10.1039/b906447h

[9] He HJ, Pires R, Zhu TN, Zhou A, Gaigalas AK, et al (2007) Fluorescence resonance energy transfer-based method for detection of DNA binding activities of nuclear factor kappaB. Biotechniques 43: 93–98. 17695258 10.2144/000112475

[10] Chen Z, Ji M, Hou P, Lu Z (2006) Exo-Dye-based assay for rapid, inexpensive, and sensitive detection of DNA-binding proteins. Biochem Biophys Res Commun 345: 1254–1263. 16716262 10.1016/j.bbrc.2006.05.012

[11] Wang J, Li T, Guo X, Lu Z (2005) Exonuclease III protection assay with FRET probe for detecting DNA-binding proteins. Nucleic Acids Res 33: e23. 15687381 10.1093/nar/gni021 PMC548379

[12] Kobayashi T, Yoshimori A, Kino K, Komori R, Miyazawa H, et al (2009) A new small molecule that directly inhibits the DNA binding of NF-κB. Bioorg Med Chem 17: 5293–5297. 19539480 10.1016/j.bmc.2009.05.030

[13] Kobayashi T, Okamoto N, Sawasaki T, Endo Y (2004) Detection of protein-DNA interactions in crude cellular extracts by fluorescence correlation spectroscopy. Anal Biochem 332: 58–66. 15301949 10.1016/j.ab.2004.05.053

[14] Rigler R (1995) Fluorescence correlations, single molecule detection and large number screening. Applications in biotechnology. J Biotechnol 41: 177–186. 7544589 10.1016/0168-1656(95)00054-t

[15] Haustein E, Schwille P (2003) Ultrasensitive investigations of biological systems by fluorescence correlation spectroscopy. Methods 29: 153–166. 12606221 10.1016/s1046-2023(02)00306-7

[16] Wolcke J, Reimann M, Klumpp M, Gohler T, Kim E, et al (2003) Analysis of p53 “latency” and “activation” by fluorescence correlation spectroscopy. Evidence for different modes of high affinity DNA binding. J Biol Chem 278: 32587–32595. 12813031 10.1074/jbc.M303615200

[17] Kinjo M, Rigler R (1995) Ultrasensitive hybridization analysis using fluorescence correlation spectroscopy. Nucleic Acids Res 23: 1795–1799. 7784185 10.1093/nar/23.10.1795 PMC306938

[18] Bone RC, Balk RA, Cerra FB, Dellinger RP, Fein AM, et al (1992) Definitions for sepsis and organ failure and guidelines for the use of innovative therapies in sepsis. The ACCP/SCCM Consensus Conference Committee. American College of Chest Physicians/Society of Critical Care Medicine. Chest 101: 1644–1655. 1303622 10.1378/chest.101.6.1644

[19] Pack C-G, Nishimura G, Tamura M, Aoki K, Taguchi H, et al (1999) Analysis of interaction between chaperonin GroEL and its substrate using fluorescence correlation spectroscopy. Cytometry 36: 247–253. 10404975 10.1002/(sici)1097-0320(19990701)36:3<247::aid-cyto15>3.3.co;2-r

[20] Michelman-Ribeiro A, Mazza D, Rosales T, Stasevich TJ, Boukari H, et al (2009) Direct measurement of association and dissociation rates of DNA binding in live cells by fluorescence correlation spectroscopy. Biophys J 97: 337–346. 19580772 10.1016/j.bpj.2009.04.027 PMC2711375

[21] Eydeler K, Magbanua E, Werner A, Ziegelmuller P, Hahn U (2009) Fluorophore binding aptamers as a tool for RNA visualization. Biophys J 96: 3703–3707. 19413975 10.1016/j.bpj.2009.01.041 PMC2711399

[22] Nomura Y, Nakamura T, Feng Z, Kinjo M (2007) Direct quantification of gene expression using fluorescence correlation spectroscopy. Curr Pharm Biotechnol 8: 286–290. 17979726 10.2174/138920107782109958

[23] Korn K, Gardellin P, Liao B, Amacker M, Bergstrom A, et al (2003) Gene expression analysis using single molecule detection. Nucleic Acids Res 31: e89. 12907741 10.1093/nar/gng089 PMC169981

[24] Xu H, Frank J, Trier U, Hammer S, Schroder W, et al (2001) Interaction of fluorescence labeled single-stranded DNA with hexameric DNA-helicase RepA: a photon and fluorescence correlation spectroscopy study. Biochemistry 40: 7211–7218. 11401568 10.1021/bi001543o

[25] Kinjo M, Nishimura G, Koyama T, Mets, Rigler R (1998) Single-molecule analysis of restriction DNA fragments using fluorescence correlation spectroscopy. Anal Biochem 260: 166–172. 9657874 10.1006/abio.1998.2652

[26] Mikuni S, Tamura M, Kinjo M (2007) Analysis of intranuclear binding process of glucocorticoid receptor using fluorescence correlation spectroscopy. FEBS Letters 581: 389–393. 17239375 10.1016/j.febslet.2006.12.038

[27] Bhattacharya N, Sarno A, Idler IS, Führer M, Zenz T, et al (2010) High-throughput detection of nuclear factor-kappaB activity using a sensitive oligo-based chemiluminescent enzyme-linked immunosorbent assay. International Journal of Cancer 127: 404–411. 19924814 10.1002/ijc.25054

[28] Jin S, Lu D, Ye S, Ye H, Zhu L, et al (2005) A simplified probe preparation for ELISA-based NF-κB activity assay. J Biochem Biophys Methods 65: 20–29. 16198424 10.1016/j.jbbm.2005.08.006

[29] Gubler ML, Abarzua P (1995) Nonradioactive assay for sequence-specific DNA binding proteins. Biotechniques 18: 1008, 1011–1004. 7546700

[30] Mokart D, Merlin M, Sannini A, Brun JP, Delpero JR, et al (2005) Procalcitonin, interleukin 6 and systemic inflammatory response syndrome (SIRS): early markers of postoperative sepsis after major surgery. British Journal of Anaesthesia 94: 767–773. 15849208 10.1093/bja/aei143

